# Exploring the determinants of ethnic differences in insulin clearance between men of Black African and White European ethnicity

**DOI:** 10.1007/s00592-021-01809-4

**Published:** 2021-10-18

**Authors:** Meera Ladwa, Oluwatoyosi Bello, Olah Hakim, Maria Linda Boselli, Fariba Shojaee-Moradie, A. Margot Umpleby, Janet Peacock, Stephanie A. Amiel, Riccardo C. Bonadonna, Louise M. Goff

**Affiliations:** 1 Diabetes Research Group, Department of Nutritional Sciences, Faculty of Life Sciences and Medicine, School of Life Course Sciences, King’s College London, Franklin-Wilkins Building, Room 3.87, Waterloo Campus, London, SE1 9NH UK; 2 Division of Endocrinology and Metabolic Disease, University of Verona School of Medicine, Verona, Italy; 3 Faculty of Health and Medical Sciences, University of Surrey, Guildford, UK; 4 Department of Epidemiology, Geisel School of Medicine, Dartmouth College, Hanover, NH USA; 5 Department of Medicine and Surgery, University of Parma and Azienda Ospedaliera Universitaria di Parma, Parma, Italy

**Keywords:** Insulin clearance, Adiponectin, Ethnicity, African, Type 2 diabetes, Inflammation

## Abstract

**Aim:**

People of Black African ancestry, who are known to be at disproportionately high risk of type 2 diabetes (T2D), typically exhibit lower hepatic insulin clearance compared with White Europeans. However, the mechanisms underlying this metabolic characteristic are poorly understood. We explored whether low insulin clearance in Black African (BA) men could be explained by insulin resistance, subclinical inflammation or adiponectin concentrations.

**Methods:**

BA and White European (WE) men, categorised as either normal glucose tolerant (NGT) or with T2D, were recruited to undergo the following: a mixed meal tolerance test with C-peptide modelling to determine endogenous insulin clearance; fasting serum adiponectin and cytokine profiles; a hyperinsulinaemic–euglycaemic clamp to measure whole-body insulin sensitivity; and magnetic resonance imaging to quantify visceral adipose tissue.

**Results:**

Forty BA (20 NGT and 20 T2D) and 41 WE (23 NGT and 18 T2D) men were studied. BA men had significantly lower insulin clearance (*P* = 0.011) and lower plasma adiponectin (*P* = 0.031) compared with WE men. In multiple regression analysis, ethnicity, insulin sensitivity and plasma adiponectin were independent predictors of insulin clearance, while age, visceral adiposity and tumour necrosis factor alpha (TNF-*α*) did not significantly contribute to the variation.

**Conclusion:**

These data suggest that adiponectin may play a direct role in the upregulation of insulin clearance beyond its insulin-sensitising properties.

## Introduction

A consistently recognised feature of physiology in Black African (BA) populations is low insulin clearance in comparison with White Europeans (WE), with studies indicating up to 74% lower insulin clearance in BA compared with WE subjects [1]. Modelling methods have found that this difference lies in hepatic, as opposed to extra-hepatic, insulin clearance [2].

Hepatic insulin clearance is predominantly mediated by binding of insulin to its receptor [3]; therefore, it is an integral part of insulin’s action on the liver. Greater insulin sensitivity has been associated with greater clearance [4], including in BA populations [5]. Accordingly, as there is evidence that BA populations may be more insulin resistant compared with other ethnicities [6], it is believed that impaired insulin sensitivity drives their low insulin clearance [7].

However, an emerging counterargument proposes that low hepatic insulin clearance may not be purely compensatory, but a primary abnormality in the development of type 2 diabetes (T2D) [8, 9]. According to this hypothesis, defects in hepatic insulin clearance promote chronic hyperinsulinaemia, which in turn leads to insulin receptor desensitisation, target tissue insulin resistance and subsequent glucose intolerance. In this model, low hepatic clearance in certain ethnic groups is the a priori risk factor underlying their increased prevalence of T2D and precedes the development of insulin resistance. Supporting this hypothesis, insulin clearance has been identified as an important predictor of T2D development in longitudinal studies of African-Americans [10].

Thus, there is considerable interest in understanding the mechanisms underlying low insulin clearance in BA populations. If it occurs independently of insulin resistance, then alternative explanations must be sought. Chronic, low-grade inflammation may be a factor, with evidence that elevated C-reactive protein (CRP) [11], white cell counts [12] and plasminogen activator inhibitor 1 (PAI-1) levels [13] are associated with impaired insulin clearance. Ethnic differences in inflammatory profiles have been recognised, including higher levels of C-reactive protein and interferon gamma (IFN-ɣ) in BA subjects [14, 15]; therefore, it is plausible that subclinical inflammation may account for the variation in hepatic insulin clearance rates observed in different ethnic groups.

Another possibility is that low insulin clearance is secondary to ethnic differences in concentrations of adiponectin, an adipokine which has numerous roles in glucose and lipid metabolism. Studies have consistently shown that BA subjects have lower plasma adiponectin levels compared with WE [16–18]. An association between adiponectin and hepatic insulin clearance has also been observed [19, 20], and the upregulation of liver adiponectin receptors is associated with greater hepatic insulin clearance in animal models [21]. However, to date, no studies have looked at the role of adiponectin in relation to insulin clearance in BA populations.

Therefore, the aim of this study was to assess ethnic differences in insulin clearance and explore its predictors—particularly insulin sensitivity, inflammatory biomarkers and adiponectin—in BA and WE men with normal glucose tolerance (NGT) and early T2D in a secondary analysis of the *SouL-DeEP* study [22].

## Materials and methods

The data were collected as part of the South London Ethnicity and Diabetes Phenotyping study *(SouL-DeEP*). The *SouL-DeEP* study was an observational cross-sectional study aimed at exploring ethnic differences in a range of mechanisms underlying the development of T2D between men of WE and BA ethnicity with NGT and early T2D. The study was designed to enable the exploration of a wide range of hypotheses, several of which have been published [22–24]*.* Data collection took place between April 2013 and April 2019. The full protocol has been published [22].

Recruitment was carried out through advertising in local press and via primary care. Eligible participants were male, 18–65 years of age and with a body mass index (BMI) of 20–40 kg/m^2^. Participants self-identified as either WE or BA. WE participants had four self-declared European grandparents with at least two of them of north-west European ancestry. BA participants had four self-declared grandparents from West African countries.

Eligible NGT participants had a fasting venous plasma glucose of < 6.1 mmol/L and a 2-h OGTT glucose of < 7.8 mmol/L. Eligible T2D participants had a diagnosis of T2D with a duration of ≤ 5 years, treated with lifestyle measures and/or metformin monotherapy and a screening HbA1c ≤ 64 mmol/mol (8.0%). Exclusion criteria were: treated with other diabetes medications, steroids or beta-blockers; serum creatinine > 150 mmol/l; serum alanine transaminase level > 2.5-fold above the upper limit of the reference range; positive auto-antibodies for insulin, glutamic acid carboxylase (GAD) or islet antigen 2 (IA2); sickle cell disease (trait permitted); or any contraindication to magnetic resonance imaging.

### Endogenous insulin clearance

Following an overnight fast, participants underwent a mixed meal tolerance test (MMTT). A cannula was inserted into an antecubital fossa vein; following sampling of fasting blood at − 10 and 0 min, participants consumed a specified volume of Ensure Plus milkshake drink (63% carbohydrate, 22% protein and 15% fat) (Abbott Nutrition, UK) based on 6 cal (4 ml) per kg body weight within a 5-min period. Further samples were drawn at 10, 20, 30, 40, 50, 60, 75, 90, 120, 150 and 180 min for assessment of glucose, C-peptide and insulin concentrations. The glucose and C-peptide curves were modelled to determine area under the curve of total endogenous insulin secretion over the 180-min duration (AUC_ISR_) [22], using SAAM-II 1.2 software (SAAM Institute, Seattle, Washington). Mean endogenous insulin clearance was then calculated according to the following formula:

$${\text{Clearance}}_{{{\text{Ins}}}} = \frac{{{\text{AUC}}_{{{\text{ISR}}}} }}{{{\text{AUC}}_{I} + (I_{{{\text{Final}}}} - I_{{{\text{Basal}}}} ) \cdot {\text{MRT}}_{{{\text{Ins}}}} }}$$

where AUC_I_ = area under the curve of insulin concentration, I_Final_ = insulin concentration at end of study, I_Basal_ = insulin concentration at start of study, MRT_ins_ = mean residence time of insulin.

For the purposes of this calculation, the MRT_ins_ is taken as 18 min for non-diabetics and 27 min for diabetics as described in Navalesi et al. [25].

### Whole-body insulin sensitivity

A 240-min two-step hyperinsulinaemic–euglycaemic clamp was conducted. Euglycaemia (5.0 mmol/l) was achieved using variable rate 20% (wt/vol) dextrose. Whole-body insulin sensitivity was assessed in the high-dose insulin phase: 40 mU m^−2^ BSA min^−1^. Blood was drawn at 150, 180, 210, 220, 230 and 240 min for the assessment of plasma glucose and insulin concentrations. Insulin sensitivity was quantified using the M value (mg m^−2^ BSA min ^−1^), which was measured during the final 30 min of the high-dose insulin phase of the clamp, calculated as total glucose disposal corrected for deviations in plasma glucose concentration. Whole-body insulin sensitivity was then expressed as M/I, the M value corrected for the steady-state insulin concentration during the last 30 min of the clamp (mg m^−2^ BSA min ^−1^)/ (pmol L^−1^).

### Visceral fat

A Dixon-based MRI sequence was used on a 1.5-Tesla Siemens scanner to obtain images from the neck to the knee (excluding arms). Three hundred and eighty-four contiguous, axial T1-weighted gradient-echo images with a slice thickness of 3 mm were acquired, from which fat and water images were produced as part of the Dixon sequence. MRI data were analysed using the open-source image analysis software HOROS V 1.1.7 (www.horosproject.org; accessed 21/10/2017) by a single analyst who was blinded to clinical data. Areas of visceral adipose tissue (VAT) were quantified from an axial MRI image at the L4-5 spinal anatomical position.

Assessments were performed in random order at the Clinical Research Facility, King’s College Hospital, London, UK, while MRI imaging took place at Guy’s Hospital, London, UK, with at least 7 days between visits.

### Laboratory analysis

Plasma adipokines and cytokines were measured on fasting plasma taken during the MMTT assessment using immunoassays (Affinity Biomarker Labs, UK). Plasma total adiponectin was measured using Human Quantikine enzyme-linked immunosorbent assay (ELISA) kits (Bio-Techne, USA). Plasma TNF-α, IFN-γ, IL-6, IL-8, IL-10 and vascular endothelial growth factor (VEGF) were determined by electrochemiluminescence using a Human Proinflammatory multiplex immunoassay, Mesoscale Quickplex Discovery SQ120 (Meso Scale Discovery, USA). Plasma glucose concentrations were determined in duplicate using an automated glucose analyzer (Yellow Spring Instruments, 2300 STAT Glucose Analyzer, Ohio, USA). Plasma insulin concentrations were determined by immunoassay using chemiluminescent technology (ADVIA Centaur System, Siemens Healthcare Ltd., Camberley, UK). Plasma C-peptide concentrations were determined by radioimmunoassay (Millipore Ltd., Hertfordshire, UK).

### Statistics

The study is a secondary analysis of the *SouL-DeEP* study which included 20 samples per ethnic group with type 2 diabetes to allow the detection of a difference of one standard deviation with power 90% and two-sided statistical significance 5% in the primary outcome variable (first-phase insulin secretion determined from the hyperglycaemic clamp test).

Normality of continuous variables was determined by inspection of histograms and Shapiro–Wilkes test. Where variables significantly deviated from normality, log transformation was carried out to achieve a normal distribution prior to the use of parametric tests. Categorical variables were analysed using Fisher’s exact test for ordinal data. A two-way between-group ANOVA was used to analyse the dependent variables of ethnicity and glucose tolerance on the outcome measures. Relationships between variables of interest were assessed with Pearson’s correlation coefficient. Multiple linear regression models were built step-wise to explore the determinant of insulin clearance with covariates of ethnicity, age, BMI, insulin sensitivity, visceral fat, adiponectin and TNF-α concentrations. As this study was a secondary analysis and exploratory, we have not adjusted for multiple testing. All analyses were conducted with SPSS, version 25.0.

## Results

The clinical characteristics of the 40 BA (20 NGT and 20 T2D) and 41 WE (23 NGT and 18 T2D) participants are presented in Table 1. The ethnic groups were similar overall in mean age, BMI, fasting glucose and HbA1c.

**Table 1:** Characteristics of study participants

	BA			WE			*P* (ethnicity)	*P* (glucose tolerance)
	All	NGT	T2D	All	NGT	T2D		
	(*n* = 40)	(*n* = 20)	(*n* = 20)	(*n* = 41)	(*n* = 23)	(*n* = 18)		
Age (years)	43.4 (15.0)	32.4 (12.1)	54.4 (7.7)	44.4 (15.1)	35.9 (13.9)	55.8 (6.8)	0.307	< 0.001
Weight (kg)	88.7 (13.0)	85.0 (13.4)	92.4 (11.8)	92.2 (17.9)	86.5 (16.5)	99.8 (17.2)	0.182	0.003
BMI (kg/m^2^)	28.4 (3.8)	26.9 (3.5)	30.0 (3.5)	28.5 (5.0)	26.5 (4.6)	31.3 (4.2)	0.612	< 0.001
Fasting glucose (mmol/ L)	5.9 (1.1)	5.1 (0.5)	6.7 (1.0)	6.0 (1.3)	5.2 (0.4)	7.0 (1.3)	0.381	< 0.001
HbA1c IFCC (mmol/mol)	44.0 (9.2)	37.5 (5.2)	50.4 (7.8)	41.6 (8.6)	35.9 (2.9)	49.2 (7.8)	0.321	< 0.001
HbA1c (DCCT) (%)	6.2 (0.8)	5.6 (0.5)	6.8 (0.7)	6.0 (0.8)	5.4 (0.2)	6.7 (0.7)	0.340	< 0.001
Systolic blood pressure (mmHg)	129.9 (15.3)	122.3 (13.0)	137.5 (13.7)	126.1 (12.4)	121.9 (9.1)	131.6 (14.3)	0.271	< 0.001
Diastolic blood pressure (mmHg)	78.9 (12.3)	71.7 (12.0)	86.1 (7.4)	75.9 (10.4)	71.1 (8.2)	82.4 (9.7)	0.325	< 0.001
LDL cholesterol (mmol/L)	2.51 (0.70)	2.71 (0.81)	2.32 (0.53)	2.70 (0.82)	2.99 (0.82)	2.31 (0.67)	0.403	0.002
HDL cholesterol (mmol/L)	1.25 (0.41)	1.32 (0.45)	1.18 (0.37)	1.24 (0.29)	1.27 (0.31)	1.21 (0.25)	0.903	0.196
Total cholesterol (mmol/L)	4.22 (0.88)	4.36 (1.03)	4.09 (0.70)	4.56 (0.94)	4.76 (1.05)	4.29 (0.72)	0.137	0.071
Triglycerides (mmol/L)	1.01 (0.62)	0.72 (0.25)	1.30 (0.74)	1.36 (0.70)	1.10 (0.56)	1.71 (0.73)	0.004	< 0.001
2-h OGTT glucose (mmol/L)	–	5.2 (1.1)	–	–	5.1 (1.3)	–	0.727	–
Duration of T2D (years)	–	–	2.73 (1.3)	–	–	3.10 (0.98)	0.298	–
Treatment with metformin (%)	–	–	70	–	–	55	0.320	–

Data presented as mean (SD)

A two-way between-group ANOVA was used to analy**s**e the dependent variables of ethnicity and glucose tolerance on the clinical characteristics

*BA* Black African, *WE* White European, *NGT* normal glucose tolerance, *T2D* type 2 diabetes, *BMI* body mass index, *DCCT* Diabetes Control and Complications Trial, *HbA1c* glycated haemoglobin, *HDL* high-density lipoprotein, *IFCC* International Federation of Clinical Chemistry, *LDL* low-density lipoprotein, *OGTT* oral glucose tolerance test

**Fig. 1:**
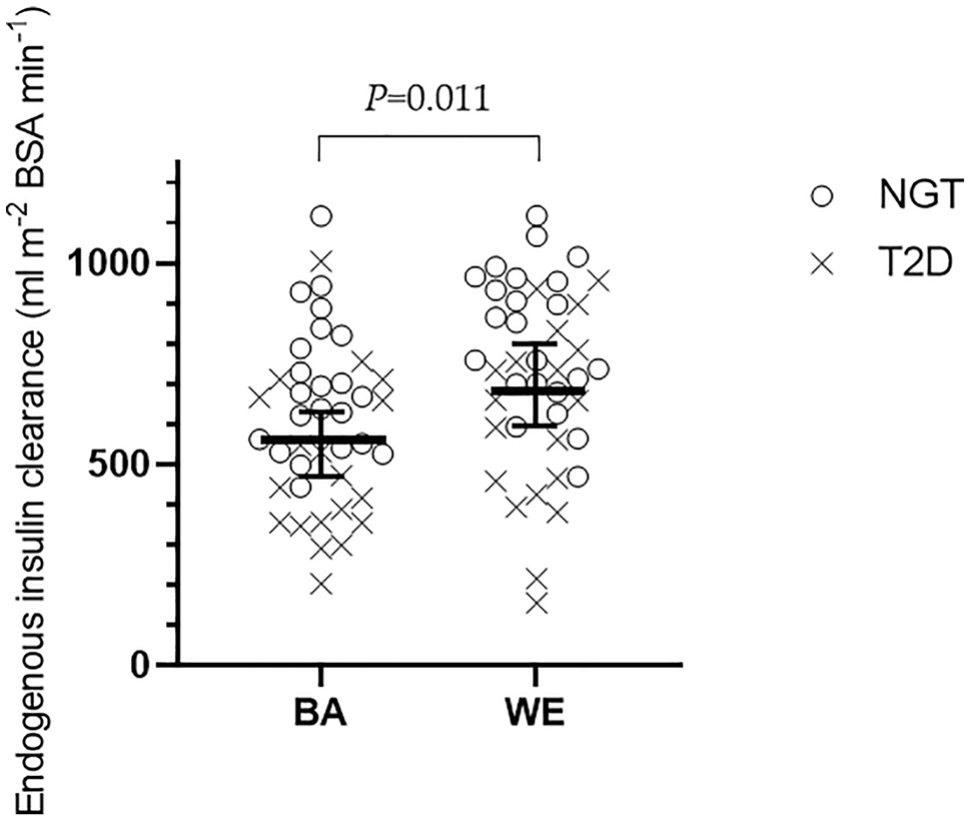
Endogenous insulin clearance by ethnicity and glucose tolerance status. BA, Black African; WE, white European; NGT, normal glucose tolerance; T2D, type 2 diabetes. Data shown as median (95% confidence intervals)

Insulin clearance, insulin sensitivity, adiponectin and inflammatory biomarker data, by ethnic group and glucose tolerance status, are presented in Table 2. There were no significant ethnic differences in insulin sensitivity across either glucose tolerance group. BA men had significantly lower mean endogenous insulin clearance (Fig. 1), plasma adiponectin and visceral adipose tissue compared with WE men. Mean IL-10 was higher in BA men. There were no significant ethnic differences in plasma TNF-α, IFN-ɣ, IL-6, IL-8 or vascular endothelial growth factor (VEGF) concentrations. Men with T2D had lower mean insulin sensitivity, insulin clearance and adiponectin but higher VAT, TNF-α, IL-8 and VEGF concentrations compared with NGT men.

**Table 2:** Metabolic measurements of participants by ethnicity and glucose tolerance

	BA			WE			*P* (ethnicity)	*P* (gluc tolerance)
	All	NGT	T2D	All	NGT	T2D		
	(*n* = 40)	(*n* = 20)	(*n* = 20)	(*n* = 41)	(*n* = 23)	(*n* = 18)		
Insulin sensitivity (mg m^−2^ BSA min^−1^)/(pmol L^−1^) †	0.41 (0.32, 0.52)	0.53 (0.45, 0.62)	0.31 (0.21, 0.40)	0.39 (0.30, 0.51)	0.53 (0.41, 0.69)	0.24 (0.17, 0.34)	0.413	< 0.001
Insulin clearance (mL m^−2^ BSA min^−1^) †	556.1 (470.8, 657.1)	681.1 (601.3, 771.4)	476.1 (394.9, 574.0)	686.6 (593.8, 794.0)	800.0 (725.9, 881.5)	568.9 (430.3, 752.0)	0.011	< 0.001
VAT (cm^2^) †	66.2 (46.8, 66.2)	46.9 (34.2, 64.3)	121.0 (100.3, 146.0)	108.3 (81.6, 143.7)	79.0 (55.4, 112.5)	184.4 (148.3, 229.3)	0.001	< 0.001
Adiponectin (mg/L) †	2.54 (1.90, 3.40)	3.18 (2.42, 4.18)	2.07 (1.57, 2.72)	3.62 (3.05, 4.32)	4.39 (3.72,5.20)	2.73 (1.99, 3.73)	0.031	0.001
TNF-α (ng/L)†	2.61 (2.42, 2.81)	2.26 (1.92, 2.66)	2.76 (2.14, 3.55)	2.74 (2.21, 3.40)	2.43 (2.20, 2.67)	2.91 (2.54, 3.33)	0.481	0.016
IFN-ɣ (ng/L)†	4.81 (3.88, 5.96)	4.85 (4.02, 5.84)	4.08 (2.59, 6.42)	4.85 (4.16, 5.66)	4.29 (3.62,5.07)	5.98 (4.44, 8.04)	0.328	0.576
IL-6 (ng/L)†	0.93 (0.80, 1.10)	0.90 (0.72, 1.12)	1.02 (0.77, 1.35)	0.96 (0.81, 1.14)	0.88 (0.71, 1.09)	1.12 (0.86, 1.44)	0.901	0.087
IL-8 (ng/L)†	8.51 (7.18, 10.09)	7.55 (6.58, 8.67)	10.55 (7.77, 14.32)	8.31 (7.21, 9.58)	7.99 (6.82,9.35)	8.88 (6.54, 12.07)	0.479	0.023
IL-10 (ng/L)†	0.74 (0.58, 1.00)	0.75 (0.62, 0.92)	0.72 (0.42, 1.25)	0.50 (0.44, 0.58)	0.53 (0.44, 0.63)	0.47 (0.36, 0.61)	0.004	0.250
VEGF (ng/L)†	79.2 (59.4, 105.6)	62.1 (42.6, 90.6)	104.5 (65.9, 165.5)	62.8 (49.2, 80.1)	51.8 (37.4, 71.7)	86.8 (62.3, 120.9)	0.560	0.002

All biomarkers measured during fasting

Data presented as mean (SD) or geometric mean (95% CI) for logged data

†BSA, body surface area. IL, interleukin; VAT, visceral adipose tissue; VEGF, vascular endothelial growth factor; TNF-α, tumour necrosis factor alpha; IFN-ɣ, interferon gamma. Data from subjects included in this study have been previously published in [22–24]. A two-way between-group ANOVA was used to analy**s**e the dependent variables of ethnicity and glucose tolerance on the outcome measures

Associations between insulin clearance and the adipocytokines for each ethnic group and the cohort as a whole are shown in Table 3. Plasma adiponectin was significantly correlated with insulin clearance in the cohort as a whole (*r* = 0.46, *P* < 0.001), but when examined within ethnicity, the association was statistically significant in only BA men. TNF-α was inversely correlated with insulin clearance in the cohort as a whole (*r* = − 0.24, *P* = 0.037), but when examined within ethnicity, this was significant in only WE men (Table 3). There were no significant relationships found between insulin clearance and IFN-ɣ, IL-6, Il-8, IL-10 or VEGF (Table 3). Multiple regression analysis showed that both ethnicity and adiponectin were significant predictors of insulin clearance in a mutually adjusted model that also included insulin sensitivity and BMI as covariates. In that model, the adjusted mean difference in insulin clearance in WE vs BA was 96 mL m^−2^ BSA min^−1^ (Table 4, Model 1*).*

**Table 3:** Relationships between insulin clearance and adipocytokines using Pearson’s correlation

	BA (*n* = 40)	WE (*n* = 41)	All (*n* = 81)
Adiponectin (mg/L)	*r* = 0.53, *P* = 0.001	*r* = 0.31, *P* = 0.059	*r* = 0.46, *P* < 0.001
TNF-α (ng/L)	*r* = − 0.17, *P* = 0.32	*r* = − 0.42, *P* = 0.008	*r* = − 0.24, *P* = 0.037
IFN-ɣ (ng/L)	*r* = 0.23, *P* = 0.27	*r* = − 0.14, *P* = 0.42	*r* = 0.04, *P* = 0.79
IL-6 (ng/L)	*r* = 0.05, *P* = 0.75	*r* = − 0.26, *P* = 0.10	*r* = − 0.10, *P* = 0.37
IL-8 (ng/L)	*r* = − 0.05, *P* = 0.75	*r* = − 0.14, *P* = 0.39	*r* = − 0.11, *P* = 0.32
IL-10 (ng/L)	*r* = 0.14, *P* = 0.41	*r* = 0.02, *P* = 0.93	*r* = 0.01, *P* = 0.95
VEGF (ng/L)	*r* = − 0.08, *P* = 0.63	*r* = 0.04, *P* = 0.83	*r* = − 0.04, *P* = 0.95

All r and *P* values for correlation with endogenous insulin clearance

**Table 4:** Multiple regression analysis of predictors of insulin clearance (mL m^−2^ BSA min^−1^)

Independent variables	Regression coefficient	95% CI	*P* value
*MODEL 1: R*^*2*^ = *0.418*			
Ethnicity (BA-WE)^a^	− 96	− 180 to − 12	0.025
Body mass index^b^	− 36.4	− 70.8 to − 2.0	0.038
Insulin sensitivity^b^	9.0	0.2–17.8	0.046
Plasma adiponectin^b^	7.9	0.5–15.3	0.036
*MODEL 2: R*^*2*^ = *0.434*			
Ethnicity (BA-WE)^a^	− 99	− 196 to − 1	0.047
Insulin sensitivity^b^	38.9	18.0–59.8	< 0.001
Plasma adiponectin^b^	20.4	2.2–38.6	0.029
Plasma TNF-α^2^	− 6.3	− 35.7 to 23.2	0.67
Age^c^	1.6	− 40.4 to 43.6	0.94
Visceral adipose tissue^b^	5.1	− 16.7 to 27.0	0.64

^a^Regression coefficient is adjusted difference in mean insulin clearance, BA-WE ^b^Variable is log-transformed. Regression coefficient and 95% CI are scaled so that each is the adjusted change in insulin clearance for a 10% increase in that variable

^c^Regression coefficient is the adjusted change in insulin clearance per 10-year increase in age

The use of visceral fat in place of BMI as a marker of adiposity, and the addition of age and plasma TNF-α as co-variates, made no appreciable difference to the estimated effects of ethnic group (99 compared to 96 mL m^−2^ BSA min^−1^). The effect of adiponectin was even greater in the fuller model and remained statistically significant (Table 4, Model 2).

## Discussion

The importance of insulin clearance as a modulator of peripheral insulin levels is increasingly recognised [26], particularly in BA populations who, in comparison with other ethnicities, have consistently been shown to exhibit a distinctly hyperinsulinaemic response to glucose, predominantly driven by relatively low insulin clearance [27]. Here, we have presented data which find that insulin clearance may be determined by ethnicity independently of insulin resistance, adiposity and subclinical inflammation and that plasma adiponectin may play a direct role in the upregulation of insulin clearance beyond its insulin-sensitising effects.

The BA men in this study exhibited the metabolic features characteristic of their ethnic group, including lower visceral fat, lower adiponectin and lower insulin clearance compared with WE men of similar age and BMI. A body of literature proposes that low insulin clearance in BA populations is secondary to insulin resistance. Lorenzo et al. presented data showing that ethnic differences in the metabolic clearance rate of insulin (MCRI) of African-American (versus Hispanic and White American) participants could be entirely explained by insulin resistance and adiposity [7]. However, in our study, by contrast, ethnicity determined insulin clearance independently of insulin sensitivity, BMI and visceral fat. The discrepancy in findings may be due to the methodologies used to measure insulin clearance. The MCRI measures the clearance of peripherally infused exogenous insulin, rather than the clearance of endogenous insulin, the latter being secreted directly into the portal vein and undergoing extraction by the liver prior to entering the systemic circulation [28]. Our measurement of insulin clearance takes account of first-pass hepatic insulin extraction, which is not captured by the MCRI. It is known that the MCRI is poorly correlated with both directly measured hepatic insulin extraction in animal models [29] and surrogate measures of hepatic insulin clearance derived from the hyperglycaemic clamp [30]. While hepatic insulin clearance is lower in BA in comparison with WE, extra-hepatic clearance is similar [2] and both these processes appear to be differentially regulated [5, 20]. Therefore, the MCRI may not be a wholly representative measure of insulin clearance when comparing these ethnic groups.

It has been argued that low insulin clearance is a primary determinant of peripheral insulin levels rather than a compensatory mechanism for insulin resistance [8, 31]. Our findings support this, as endogenous insulin clearance in the BA subjects remained low even when adjustments for insulin sensitivity were made. We have previously shown that low insulin clearance compensates for postprandial insulin secretion deficiencies in BA subjects and therefore may act as a protective mechanism [24]. However, under conditions where insulin secretion rates are excessively stimulated (such as during the hyperglycaemic clamp), low insulin clearance leads to a marked hyperinsulinaemia [27]. Chronic hyperinsulinaemia is thought to be metabolically deleterious, predisposing to insulin resistance, beta-cell stress and glucose intolerance [32]. Therefore, a metabolic characteristic which under some conditions may be an evolutionary advantage, relieving the demand on the beta cell to upregulate insulin secretion, may in other environments prove unfavourable. As this was not a longitudinal study, it cannot provide evidence of a causative relationship between low insulin clearance and T2D. However, the significantly lower insulin clearance in NGT BA men, in the presence of comparable insulin sensitivity to their WE counterparts, supports the hypothesis that impairments in insulin clearance precede the development of other metabolic abnormalities in the progression to glucose intolerance. Therefore, manipulation of hepatic insulin clearance may offer a novel therapeutic target for T2D in this high-risk ethnic population.

Much remains to be understood in relation to insulin clearance and its regulation; therefore, any interpretations of these data are necessarily speculative. Although insulin resistance does not account for low insulin clearance in BA men, we did not find that ethnic differences in cytokine admixture provided an alternative explanation. Consistent with the literature, where various inflammatory markers have been linked to impaired insulin clearance [12, 13], we found a negative correlation between TNF-*α* and insulin clearance. However, in agreement with others, this did not persist following correction for insulin sensitivity and adiposity [13]. Thus, subclinical inflammation per se does not appear to directly affect insulin clearance but accompanies it through associations with other features of metabolic dysfunction. It must be acknowledged that in a small sample, the failure to observe a relationship between two variables does not mean that a relationship is absent, and further work into the link between inflammation and insulin clearance is indicated.

On the other hand, adiponectin was associated with increased insulin clearance independently of insulin sensitivity and adiposity. Higher adiponectin concentrations are associated with a reduced risk of T2D in BA subjects [33, 34], which is thought to be mediated via adiponectin’s beneficial effects on both insulin sensitivity [34] and beta-cell proliferation and survival. However, the importance of adiponectin as an independent modulator of insulin clearance in BA populations has never been examined. Adiponectin receptors are abundantly expressed in the liver, and adiponectin has pleiotropic effects on hepatic metabolism, including suppression of hepatic glucose production and lipogenesis [35]; but adiponectin’s role in hepatic insulin clearance has been little explored. While an association between adiponectin and insulin clearance has been previously observed [19, 20], this is the first time a relationship has been demonstrated independently of adiposity and insulin sensitivity. The findings of this study suggest that an examination of adiponectin signalling and hepatic insulin clearance is warranted at a molecular level, in order to investigate whether adiponectin is an active player in the mechanisms of clearance.

While low adiponectin concentrations appear to be an important factor, they do not entirely explain the low insulin clearance of the BA men. Low insulin clearance has been demonstrated in a wide variety of BA subjects, including healthy men [27] and women [36], obese adolescents [37] and adults with T2D [38]. The highly conserved nature of this trait is well illustrated by Osei et al*.* who demonstrated that while African-Americans, native Ghanaians and Ghanaian immigrants to the USA have similar rates of insulin clearance to each other, all three BA groups have lower insulin clearance compared with White Americans [39]. The consistency of this observation in disparate BA populations, regardless of sex, age or indigenous or diasporic origin, is suggestive of an inherent rather than environmental cause, although to date no responsible genetic variant in BA populations has been identified.

While the mechanisms underlying this ethnic characteristic remain elusive, given that insulin clearance appears to be regulated in the BA men independently of insulin’s action on glucose uptake, we hypothesise that specific signalling pathways may be involved. One study has identified lower insulin-degrading enzyme (IDE) activity in the liver tissue of African-Americans [40] as a possible explanation, although the importance of IDE in the regulation of hepatic insulin clearance has been questioned [41, 42]. Other potential avenues of interest include the role of carcinoembryonic antigen-related cell adhesion molecule-1 (CEACAM-1) [28], zinc ion transport [43] and the signals which determine rates of retro-endocytosis (by which a variable proportion of receptor-bound, internalised insulin undergoes rapid exocytosis from the hepatocyte and is returned back to the circulation [44]). The findings of this study present an appeal to basic scientists to re-examine the intracellular pathways of insulin trafficking and degradation, an area which needs further exploration.

The strengths of this study are its measurement of endogenous as opposed to exogenous insulin clearance and the use of the reference-standard hyperinsulinaemic–euglycaemic clamp to measure insulin sensitivity. The use of magnetic resonance quantification of visceral adipose tissue provided a more accurate measure of adiposity than BMI alone. Furthermore, the subjects were tightly characterised, with the BA men of strict West African ancestry. In terms of limitations, the modelled measure of endogenous insulin clearance does not enable differentiation between hepatic and extra-hepatic insulin clearance. The assumption is made that whole-body clearance is a good proxy of hepatic insulin clearance, as hepatic insulin clearance is responsible for around 80% of whole-body clearance [45]. However, others have found that in BA populations, the proportion of total endogenous insulin clearance attributable to the liver may be as little as 30% [5]. The inability to distinguish between the two processes means that the specific effect of ethnicity in relation to hepatic insulin clearance—and its relationships with hepatic insulin sensitivity—could not be isolated. Our small sample size means that only large differences in the mean could be detected. Furthermore, our study is a secondary exploratory analysis, so we have not adjusted for multiple testing. Hence, our findings need replicating in other larger datasets. Finally, these findings apply to adult men only; therefore, they cannot necessarily be generalised to female or child/adolescent populations.

In conclusion, in a cohort of BA men, neither insulin resistance, inflammatory cytokines nor adiponectin concentrations appear to fully account for their low insulin clearance. We hypothesise that an inherent ethnic variation in intracellular insulin degradation pathways is responsible for this metabolic phenomenon, one that has evolved due to adaptive advantage but, in accordance with emerging hypotheses, has potentially deleterious effects on glucose tolerance in the setting of excessive beta-cell stimulation. The identification and manipulation of such pathways offer a novel avenue of investigation for the prevention and management of T2D.

## Data Availability

Data are available upon request from the corresponding author.

## Abbreviations

BA: Black African(s)
BMI: Body mass index
HbA1c: Glycated haemoglobin
IFN-ɣ: Interferon gamma
IL: Interleukin
NGT: Normal glucose tolerance
OGTT: Oral glucose tolerance test
T2D: Type 2 diabetes
TNF-α: Tumour necrosis factor alpha
VEGF: Vascular endothelial growth factor
WE: White European(s)
